# Performance analysis of a computer-aided detection system for lung nodules in CT at different slice thicknesses

**DOI:** 10.1117/1.JMI.5.1.014504

**Published:** 2018-02-19

**Authors:** Barath Narayanan Narayanan, Russell Craig Hardie, Temesguen Messay Kebede

**Affiliations:** University of Dayton, Department of Electrical and Computer Engineering, Dayton, Ohio, United States

**Keywords:** computer-aided detection, computed tomography, lung nodules, slice thickness, downsampling

## Abstract

We study the performance of a computer-aided detection (CAD) system for lung nodules in computed tomography (CT) as a function of slice thickness. In addition, we propose and compare three different training methodologies for utilizing nonhomogeneous thickness training data (i.e., composed of cases with different slice thicknesses). These methods are (1) aggregate training using the entire suite of data at their native thickness, (2) homogeneous subset training that uses only the subset of training data that matches each testing case, and (3) resampling all training and testing cases to a common thickness. We believe this study has important implications for how CT is acquired, processed, and stored. We make use of 192 CT cases acquired at a thickness of 1.25 mm and 283 cases at 2.5 mm. These data are from the publicly available Lung Nodule Analysis 2016 dataset. In our study, CAD performance at 2.5 mm is comparable with that at 1.25 mm and is much better than at higher thicknesses. Also, resampling all training and testing cases to 2.5 mm provides the best performance among the three training methods compared in terms of accuracy, memory consumption, and computational time.

## 1 Introduction

According to the National Cancer Institute, 234,030 lung and bronchus cancer cases are expected by the end of 2018.^1^ Lung cancer causes the most cancer-related deaths.^2^ Early detection of lung cancer could improve one’s survival rate, which rises to 87% if diagnosed in stage I.^3^ Formation of pulmonary nodules in a lung is often an indication of lung cancer. Early detection of nodules could improve a patient’s chance of survival with improved treatment options.^4^

At present, radiologists utilize computed tomography (CT) scans and chest radiographs to detect such lung nodules. In Ref. 4, it was shown that CT scans are effective in detecting such nodules. CT provides numerous slices of image data, especially when operated at a higher resolution (small slice thickness), which can be time-consuming and potentially fatiguing for radiologists to study. Hence, computer-aided detection (CAD) of lung nodules in CT scans would be valuable for lung cancer screening. CT imagery varies by slice thickness, scanner, reconstruction algorithm, and dosage settings.^5^ Generally, one would like to utilize the best resolution (small slice thickness) for CT scan; however, that would mean higher dosage for the patient. Also, operating CT scans at a small thickness value tends to increase the computational complexity and memory space of the CAD system. The performance of the CAD system for detection of lung nodules at 1.25- and 5-mm collimated slice thicknesses is compared in Ref. 5. In Ref. 6, the accuracy of the CAD system for detection of lung nodules using different reconstruction slice thickness protocols in multidetector CT is evaluated. The impact of slice thickness and radiation dosage levels for CAD of lung nodules in CT scans is presented in Ref. 7. A noise addition model is developed to simulate various dosage levels. In addition, the data are reconstructed using a medium sharp kernel at slice thicknesses of 1.5 and 3 mm. The study is conducted for 7 cases with a total of 28 radiologists markings. Hence, we believe a study of CAD performance as a function of slice thickness for a larger pool of cases and a study of methods for managing nonhomogeneous thickness training data are valuable for CAD systems.

Several research papers have been published in the field of CAD of lung nodules^6^^–^^38^ in various modalities. A CAD system developed by two of the current authors to detect lung nodules in CT scans is presented in Ref. 8. In that paper, intensity-based thresholding along with morphological processing is utilized to detect and segment the candidates simultaneously. A set of 245 features is computed for every potential nodule candidate, and they are classified as nodules or nonnodules using a Fisher linear discriminant (FLD) classifier. In Ref. 9, a CAD system to detect nodules in chest radiographs is presented. An “N-Quoit filter” is utilized in Ref. 10 for automated detection of lung nodules. Fuzzy clustering-based diagnosis rules are described in Ref. 11. The algorithm proposed in Ref. 12 combines 2-D and 3-D feature analysis using a linear discriminant classifier for CAD of lung nodules. A template-matching technique using a genetic algorithm is proposed in Ref. 13. A simple rule-based classifier to attenuate false positive (FP) findings is presented in Ref. 14. The initial validation and implementation of deep learning in CAD systems for pulmonary nodule detection and diagnosis are provided in Ref. 15. An optimized feature selection-based clustering approach for CAD of lung nodules in CT scans and chest radiographs is presented in Ref. 16. An optimal suite of intensity, shape, and texture features is used for classification purposes in Ref. 17. In Ref. 18, performance of various classifiers such as support vector machine (SVM), K-nearest-neighbor, decision tree, and linear discriminant analysis (LDA) is compared. A gradient intensity feature descriptor for pulmonary nodule classification is presented in Ref. 19. In Ref. 20, random forest and SVM classifiers are compared for CAD detection of lung nodules based on 22 handcrafted features. FLD, quadratic, and linear classification techniques are compared for CAD of lung nodules in Ref. 21. Some of the other published CAD algorithms are described in Refs. 22–38.

In this paper, we address two important issues for CAD of lung nodules in CT scans. The first issue relates to how slice thickness impacts CAD performance given training and testing data of the same thickness. This experiment has implications for how CT is acquired and/or how it may be resampled for CAD processing. The second issue relates to how to train a CAD system for best performance given nonhomogeneous slice thickness training data. Generally, one would like to use all the training data available. However, this would mean pooling of CT scans obtained from a variety of scanners and acquisition parameters, such as slice thickness and dosage settings. We propose and compare three methodologies for utilizing nonhomogeneous slice thickness training data.

To study the impact of slice thickness on CAD performance, we use the following approach. We study the CAD performance at the native thickness of 1.25 mm and three other downsampled stages for the same set of training and testing cases. This study helps us determine the slice thickness at which a CT scan could be acquired for optimal CAD performance both in terms of accuracy and computational complexity. To determine the best method of training for nonhomogeneous slice thickness data, we propose and compare three methodologies. At first, we employ the traditional CAD system approach where the entire suite of data is utilized at their native thickness (aggregate training method). Later, we study a homogeneous approach where only the cases that match with the slice thickness of testing data would be utilized for training purposes. Finally, we resample all the training and testing cases to a specific thickness value and study its impact on CAD performance. The main purpose of these experiments is to study the CAD performance despite varied training compositions. All the experiments conducted in this research are implemented on the publicly available Lung Nodule Analysis (LUNA16) dataset,^38^^,^^39^ thereby setting a benchmark for future research efforts. We conduct two sets of experiments utilizing 1.25- and 2.5-mm slice thickness data from LUNA16 dataset for each of the three methodologies to validate our study.

Our study indicates that CAD performance on 2.5-mm thickness data is comparable with 1.25 mm and is significantly better than 5.0 and 10.0 mm for the same set of training and testing cases. This result suggests that the lower dose and reduced data associated with 2.5 mm may be preferable to 1.25 mm both in terms of CAD performance and processing time. Also, we find that resampling the entire suite of data, i.e., both training and testing, to a common slice thickness of 2.5 mm provides the best results in terms of accuracy, computation time, and memory consumption for the data studied in this paper.

The remainder of this paper is organized as follows. Section 2 provides a brief description of the LUNA16 database employed for this research. Section 3 presents the CAD algorithm adopted in this paper. Section 4 describes the impact of slice thickness for CAD of lung nodules. Section 5 elucidates the various training methods with nonhomogeneous data along with their experimental results. Finally, a discussion and conclusions are given in Secs. 6 and 7, respectively.

## 2 Materials

In this paper, we utilize the data presented for the LUNA16 grand challenge set up for the evaluation of CAD algorithms to detect lung nodules.^38^^,^^39^ The dataset used for the LUNA16 challenge is a subset of the Lung Image Database Consortium–Image Database Research Initiative (LIDC-IDRI) database provided at the National Biomedical Imaging Archive. This publicly available dataset in The Cancer Imaging Archive was created for the development of CAD systems in CT scans. The LIDC-IDRI data are collected from various sites within the United States.^26^ This established database was initiated by the National Cancer Institute, which was further enhanced by the Foundation of the National Institutes of Health along with Food and Drug Administration.^26^ The LIDC-IDRI dataset contains 1018 CT scans of 1010 different patients.^26^ For LUNA16 grand challenge, 888 CT scans are selected from the LIDC-IDRI database. The LUNA16 dataset contains a substantial quantity of CT scans with different slice thicknesses, which is ideal for the study conducted in this research. A panel of four radiologists studied the CT scans in the LUNA16 dataset. Four radiologists independently annotated scans and marked all the suspicious lesions. Annotations above 3 mm that were marked by at least three of the four radiologists were considered for the LUNA16 challenge. The LUNA16 grand challenge comprised 1351 nodule cue points marked by radiologists. In this research, we utilize 192 cases from the LUNA16 dataset with 268 nodule cue points with slice thickness and slice spacing of 1.25 mm. In addition, we make use of 283 cases with 322 nodule cue points marked by radiologists with slice thickness and slice spacing of 2.5 mm. These nodules cue points are distinct with no redundant radiologists’ markings. For instance, if a nodule is marked by three different radiologists, we evaluate our performance by considering it a single target nodule rather than three different markings, thereby avoiding redundant nodule markings for evaluation purposes.

## 3 CAD System Architecture

**Fig. 1:**
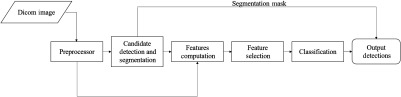
Top-level block diagram of the CAD system adopted from Ref. 8.

In this section, we describe the CAD system architecture implemented in this paper. The top-level block diagram of the overall CAD system for CT scans adopted in this paper from Ref. 8 is shown in Fig. 1. Lung segmentation is performed on the CT scans as described in Ref. 8. Nodule candidates are detected and segmented simultaneously using the method proposed in Ref. 8 using multiple gray-level thresholding. Each threshold operation is paired with a specific morphological opening operation to produce a total of 15 intermediate masks. A size- and compactness-based expert filter is later utilized to remove many unwanted intermediate candidates. A logical-OR operation is performed to obtain the final candidate mask. Further details of this algorithm can be found in Ref. 8.

After detection and segmentation of potential nodule candidates, the CAD system needs to perform a pattern recognition task. To implement this, the candidates are represented by points in feature space. Various sets of features have been proposed in the literature for the classifier to distinguish the candidates. We compute an entire suite of 503 features for all the detected potential candidates, which includes the 345 features mentioned in Ref. 8. These features include geometrical, gradient, and intensity characteristics for the raw image and various enhanced images. Details of these features are provided in Ref. 8. The 345 features are shortlisted to 245 based on linear independence in Ref. 8. We differ in this paper by shortlisting the top 300 from 503 features based on rank with the receiver operating characteristic (ROC) curve criterion.^40^ In both Refs. 8 and 9, sequential forward selection (SFS) of features is implemented to determine the optimal set of features for classification purposes solely based on the training dataset. In SFS, features are added to an empty set one by one. At each step, one feature is added, and we measure the classification performance of the system. The features that provide the best performance are selected. This type of selection helps us avoid exhaustive enumeration. The performance is measured in terms of free-response receiver operating characteristic (FROC) curve. It measures the overall sensitivity of the CAD system for a set of average number of FPs per case. In Ref. 8, the key portion to measure the area under the FROC curve (AUC) is from 0 to 10 FPs. We adopt the same criterion in this paper. The candidates are distinguished as nodules or nonnodules with the help of an FLD classifier. It has the capability to form a well-defined boundary despite uneven distribution of data.

In this paper, we report the results for the CAD system in terms of FROC analysis. We study the AUC from 0 to 10 FPs along with their confidence intervals. AUC up to a specific FP rate is a significant metric for measuring the performance.^41^ In addition, we report the Automated Nodule Detection (ANODE) 2009 scoring metric to further analyze the CAD performance. The ANODE score is defined as the average sensitivity at 7 predefined FP rates: 0.125, 0.25, 0.5, 1, 2, 4, and 8 in the FROC curve. The ANODE score was used as a metric to measure CAD performance in the ANODE 2009^24^ and LUNA16^39^ grand challenges.

## 4 Impact of Slice Thickness for CAD of Lung Nodules

### 4.1 Description of the Study

In this section, we describe a methodology to study the impact of the CAD system performance based on slice thickness of CT scans. We exclusively use 192 CT scans with slice thickness equal to slice spacing of 1.25 mm for this study. We average pairs of slices together and maintain the Hounsfield units. Averaging the densities post reconstruction provides the same average density as a thicker slice, assuming ideal reconstruction. We downsample at different ratios of 2, 4, and 8, thereby effectively achieving a simulated thickness of 2.5, 5, and 10 mm, respectively. For instance, a downsampling ratio of two is achieved by averaging two consecutive slices in a CT scan and so on. Original cue points marked by radiologists at the native slice thickness of 1.25 mm are mapped to corresponding equivalent points at different downsampled thicknesses.

We apply the candidate detector as described in Sec. 3 to determine the potential candidates at all thickness stages. We compute a set of 503 features for each candidate. We randomly pick 80 cases with 116 target nodules for testing, and the rest of the 112 CT scans are utilized for training purposes. We select features solely based on the training dataset using the SFS method, and classification of the test candidates is performed using an FLD classifier. Note that we utilize the same set of cases for testing and training at all thicknesses. This study at native and various downsampled stages helps us analyze the CAD performance at different slice thicknesses.

We measure the CAD performance at all thicknesses based on the nodule cue points marked by radiologists at 1.25 mm. This helps us compare the performance of the CAD system using the same set of nodule cue points at different thicknesses. This approach differs from existing CAD papers in which different datasets are utilized at different thicknesses for performance study. Note that we maintain the homogeneity between the train and test cases in terms of slice thickness as emphasis of this experiment is to study the performance of the CAD system at different slice thicknesses.

### 4.2 CAD Performance at Different Slice Thicknesses

**Fig. 2:**
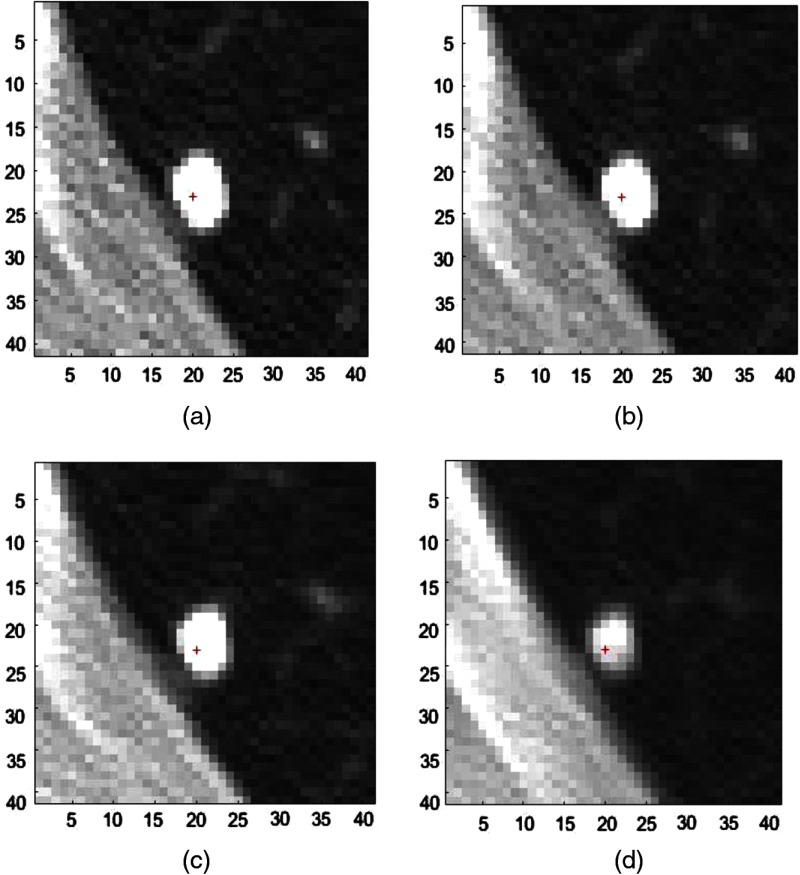
Nodule image from the case “sub0_p73” at (a) native 1.25-mm thickness, (b) simulated 2.5-mm thickness, (c) simulated 5-mm thickness, and (d) simulated 10-mm thickness.

**Fig. 3:**
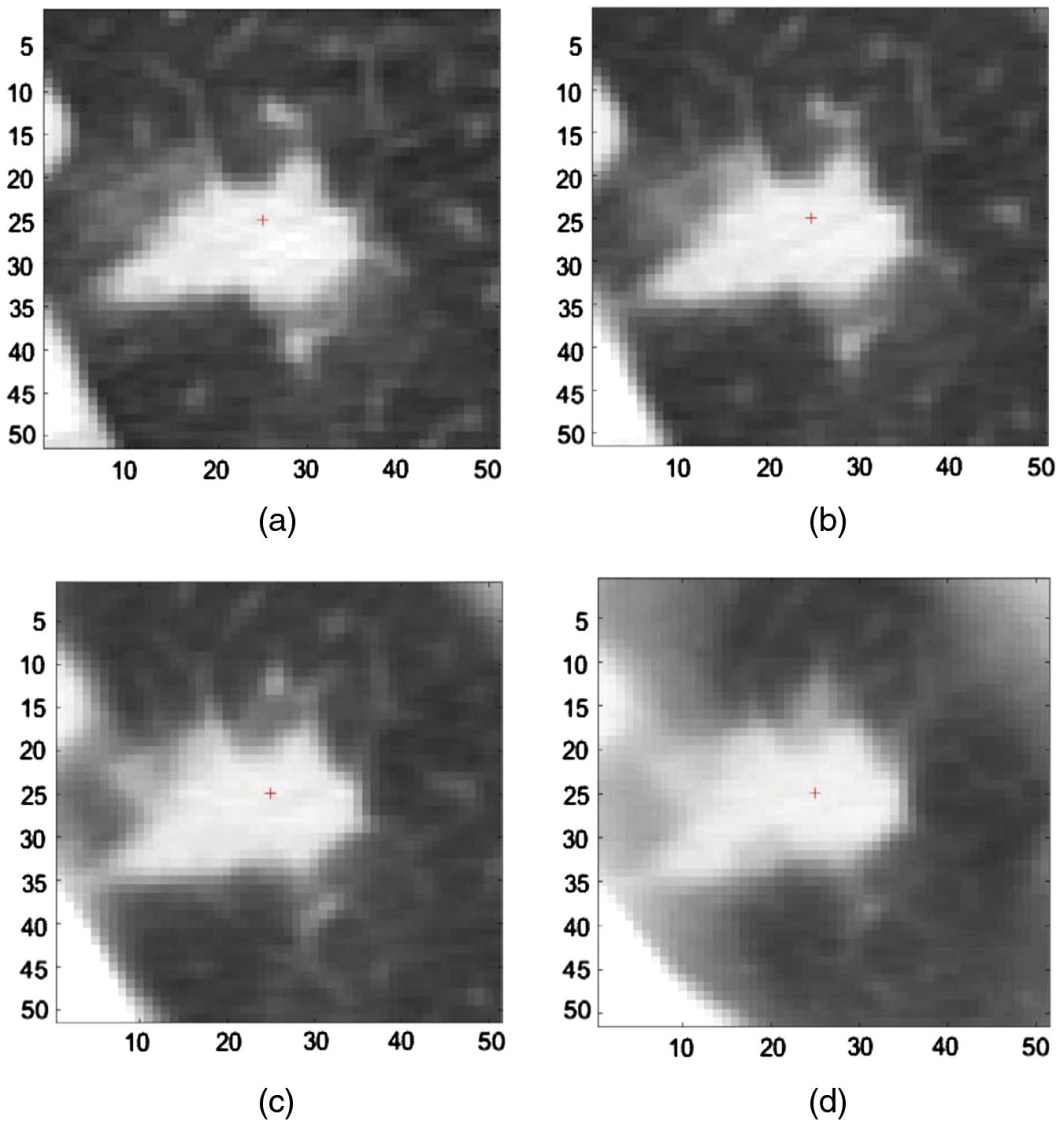
Nodule image from the case “sub0_p32” at (a) native 1.25-mm thickness, (b) simulated 2.5-mm thickness, (c) simulated 5-mm thickness, and (d) simulated 10-mm thickness.

In this section, we present results for the study presented in Sec. 4.1. Figures 2 and 3 present images of a small and large nodule at different thicknesses. Both nodule cue points have been transformed to equivalent points at different simulated thicknesses based on radiologists’ marking at 1.25-mm slice thickness. Figures 2 and 3 clearly suggest that nodules tend to lose their shape, size, and brightness at higher slice thickness, especially at 10 mm. Figures 2 and 3 also indicate that the impact of downsampling is relatively high for small nodules.

**Fig. 4:**
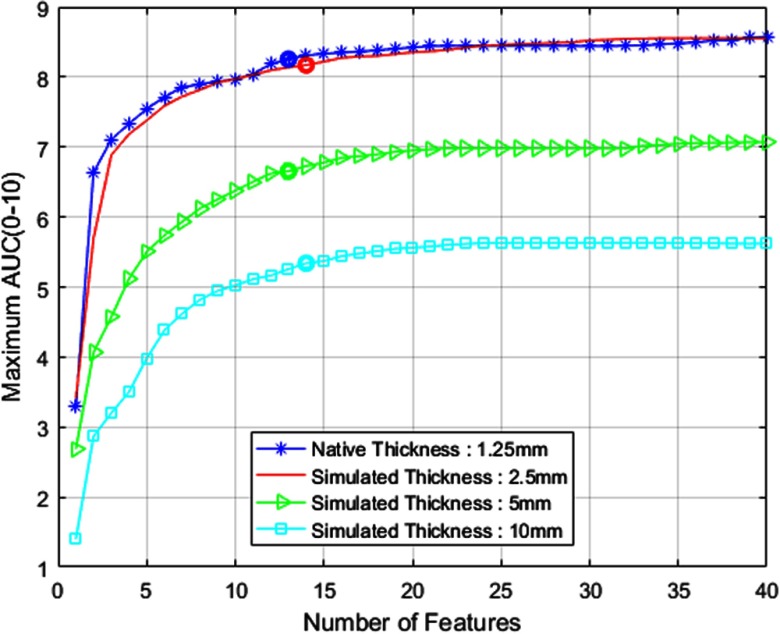
SFS merit function for 1.25-mm LUNA16 training set at all thickness levels.

**Fig. 5:**
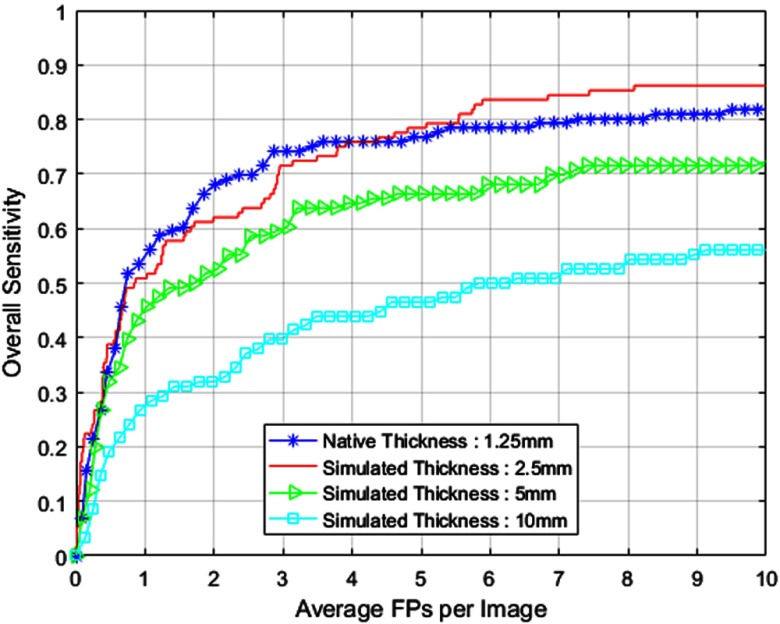
FROC curves comparing CAD performance at all thickness levels.

SFS method of feature selection is implemented at the native thickness of 1.25 mm and simulated thickness stages solely based on their respective training datasets. As mentioned earlier, the SFS merit function is measured in terms of AUC from 0 to 10 FPs. The AUC value obtained after selection of each feature is shown in Fig. 4. We choose a point in the AUC plot as implemented in Ref. 8 to determine the optimal suite of features necessary for the best classification performance. Table 1 lists the features selected (represented by X) by SFS algorithm for classification purposes at different thicknesses. The description of these features is provided in Ref. 8. Local contrast enhancement (LCE) images obtained with window size of 11 and 51 are represented by LCE1 and LCE2, respectively. We adopt this SFS approach for other experiments conducted in this study as well. Figure 5 shows the FROC curves comparing the overall CAD performance (including candidate detection and classification) at different thicknesses.

**Table 1:** Feature selected using SFS for classification using 1.25-mm LUNA16 training dataset at all thickness levels.

Feature name	1.25 mm	2.5 mm	5 mm	10 mm
Number of slices	X	—	X	—
Equivalent diameter	X	X	X	—
Periapsis	—	—	X	X
Circularity	—	X	—	—
Elongation	X	X	—	—
Minimum voxel LCE2	—	—	—	X
Standard deviation inside LCE1	—	—	X	—
Fisher ratio	—	X	—	—
Moment 1	X	X	—	—
Moment 1 LCE2	—	—	X	X
Radial-deviation mean outside	—	—	X	—
Radial-deviation standard deviation outside	—	—	—	X
Radial-gradient standard deviation outside LCE2	—	—	—	X
Standard deviation inside	—	X	—	—
Fisher ratio 1	—	—	—	X
Standard deviation separation 3	X	X	X	X
Fisher ratio 3	X	—	—	—
Fisher ratio LCE1	—	X	—	X
Contrast Z	—	—	—	X
Fisher ratio Z	—	X	X	—
Gradient magnitude mean outside 1	X	—	—	—
Radial-deviation mean outside 2	—	—	X	—
Radial-deviation mean outside 3	—	—	X	—
Radial-deviation standard deviation outside 2	—	—	—	X
Radial-deviation standard deviation outside 3	—	—	X	—
Radial-gradient perimeter standard deviation separation inside	—	X	—	—
Radial-gradient perimeter mean outside 1	X	—	—	—
Radial-gradient perimeter mean outside 2	X	—	—	—
Radial-gradient perimeter standard deviation outside 1	X	—	—	—
Radial-deviation mean inside	—	X	—	—
Radial-gradient standard deviation outside 2	—	—	—	X
Radial-deviation mean separation	—	X	—	—
Surface gradient LCE1	X	—	—	—
Area outside	X	—	—	—
Distance to center projection	X	—	X	X
Standard deviation voxel below	—	X	—	—
Standard deviation voxel below LCE1	—	—	—	X
Bottom shadow fraction	—	—	—	X
X-fraction global	—	X	X	—

Table 2 summarizes the overall CAD performance for the testing dataset utilized in this study at different thicknesses with target nodules marked by radiologists at 1.25 mm. Note that every nodule marked by the radiologists at native thickness is transformed into an equivalent cue point at all thickness stages. To determine the confidence interval, we study the performance of our CAD algorithm for the given suite of testing cases by dividing them into 10 different sets. Table 2 shows that the performance of the candidate detector before the application of the classifier is consistent at all thickness stages presented in this paper.

**Table 2:** Overall CAD performance comparison at all thickness levels.

Type of dataset (based on thickness)	Candidate detector sensitivity (before classification)	Number of features selected for classification	Overall CAD performance AUC (0 to 10 FPs)	95% Confidence AUC (0 to 10 FPs) interval	ANODE score
Native 1.25 mm	91.37	13	7.16	7.09±0.90	0.496
Simulated 2.5 mm	**91.37**	**14**	**7.29**	7.37±0.50	**0.513**
Simulated 5 mm	92.24	13	6.13	6.20±0.86	0.418
Simulated 10 mm	85.34	14	4.34	4.40±0.79	0.277

Note: Bold values represent the best performance among the compared methods.

## 5 Different Training Methods for CAD of Lung Nodules Using Nonhomogeneous Training Data

### 5.1 Description of the Methods

The aggregate training method is used in the majority of the CAD systems presented in the literature. In this method, we utilize all the training data available at their respective native thickness. CT scans are neither resampled nor removed in this approach, thereby using all the available training cases at their respective native thickness.

The homogeneous thickness training method utilizes only the cases that match with the thickness and spacing of the testing cases. For instance, if testing is conducted on cases acquired at 1.25-mm thickness, then training would be solely based on the data acquired at 1.25 mm, thereby making it a homogeneous thickness classifier.

Finally, we propose a method to maintain the homogeneity between testing and training datasets by resampling the entire suite of CT scans to a specific thickness value. This method of classification helps in utilizing all the available training resources and maintaining the homogeneity among the cases (training and testing). We term this approach the common thickness method of classification.

### 5.2 Experiment Based on 1.25-mm Testing Dataset

In this section, we present and compare results for the methodologies proposed in Sec. 5.1 for the testing cases acquired at 1.25-mm thickness. We utilize the same set of 80 cases as chosen in Sec. 4 for testing purposes. The rest of the cases available are utilized for training the CAD system. Table 3 presents the distribution of the training and testing datasets used for the three different methods of classification. Different training methodologies with different compositions are designed to reflect the real-world scenarios. A number of cases utilized for training purposes using the aggregate and common thickness training methods are always the same. We intentionally designed the homogeneous thickness training method with fewer cases because, in practice, training cases that match with thickness and spacing of a given testing case will generally be fewer in number.

**Table 3:** Training and testing dataset compositions for different methods of classification—experiment based on 1.25-mm testing dataset.

Classification approach	Training dataset (number of cases)			Testing dataset (number of cases)	
	1.25 mm	2.5 mm	1.25 to 2.5 mm	1.25 mm	1.25 to 2.5 mm
Aggregate	112	283	0	80	0
Homogeneous thickness	112	0	0	80	0
Common thickness	0	283	112	0	80

**Fig. 6:**
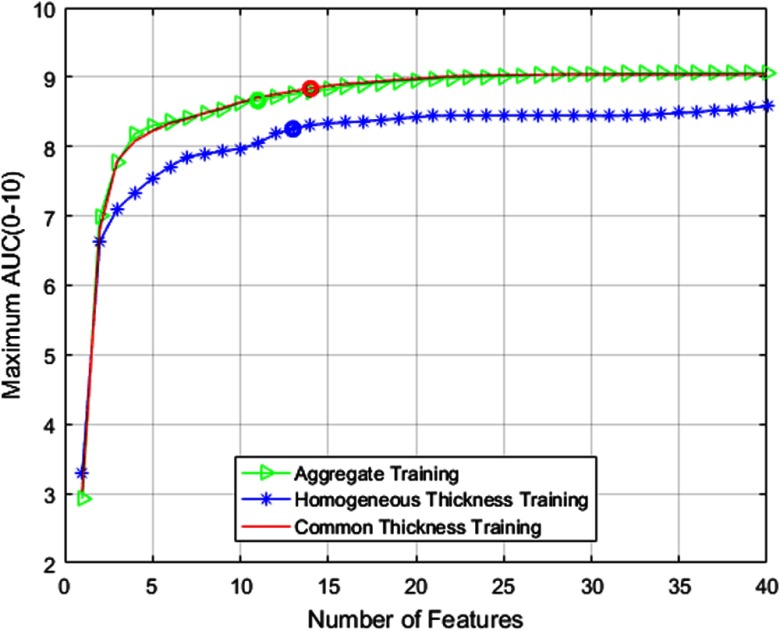
SFS merit function for different training methods.

**Fig. 7:**
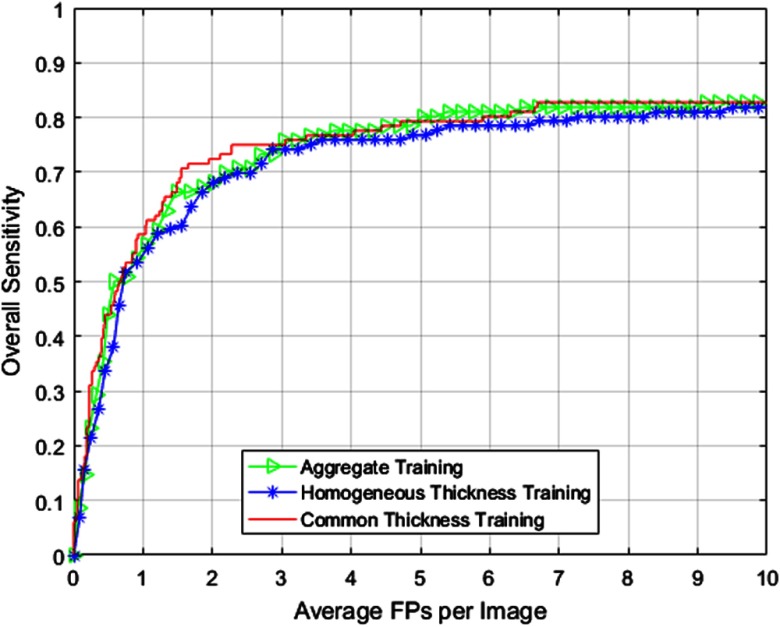
FROC curves comparing overall CAD performance using different training methods for 1.25-mm testing dataset utilizing the composition provided in Table 3.

The candidate detector (before the application of classifier) was successfully able to detect 106 of the 116 target nodules for our testing dataset at both native thickness of 1.25 mm and simulated downsampled thickness of 2.5 mm. SFS merit function plot is shown in Fig. 6. FROC results comparing the various modes of training are presented in Fig. 7. Table 4 summarizes the overall CAD performance using three different training methods. Like Sec. 4.2, to study the confidence interval, we divide the testing set into 10 different subsets.

**Table 4:** Overall CAD performance comparison using different training methods for 1.25-mm testing dataset.

Training method	Candidate detector sensitivity (before classification)	Number of features selected for classification	Overall CAD performance AUC (0 to 10 FPs)	95% Confidence AUC (0 to 10 FPs) interval	ANODE score
Aggregate	91.37	11	7.36	7.27±0.78	0.530
Homogeneous thickness	91.37	13	7.16	7.09±0.90	0.496
Common thickness	**91.37**	**14**	**7.44**	7.44±0.46	**0.544**

Note: Bold values represent the best performance among the compared methods.

### 5.3 Experiment Based on 2.5-mm Testing Dataset

We study and compare the performance of all training methods with testing being conducted on 100 cases acquired at 2.5 mm with 114 target nodules. We utilize the rest of the cases available for training purposes. The distribution of training and testing datasets for this experiment is listed in Table 5 for the classification methods proposed in Sec. 5.1.

**Table 5:** Training and testing dataset compositions for different methods of classification—experiment based on 2.5-mm testing dataset.

Classification approach	Training dataset (number of cases)			Testing dataset 2.5-mm slice thickness (number of cases)
	1.25 mm	2.5 mm	1.25 to 2.5 mm	
Aggregate	192	183	0	100
Homogeneous thickness	0	183	0	100
Common thickness	0	183	192	100

**Fig. 8:**
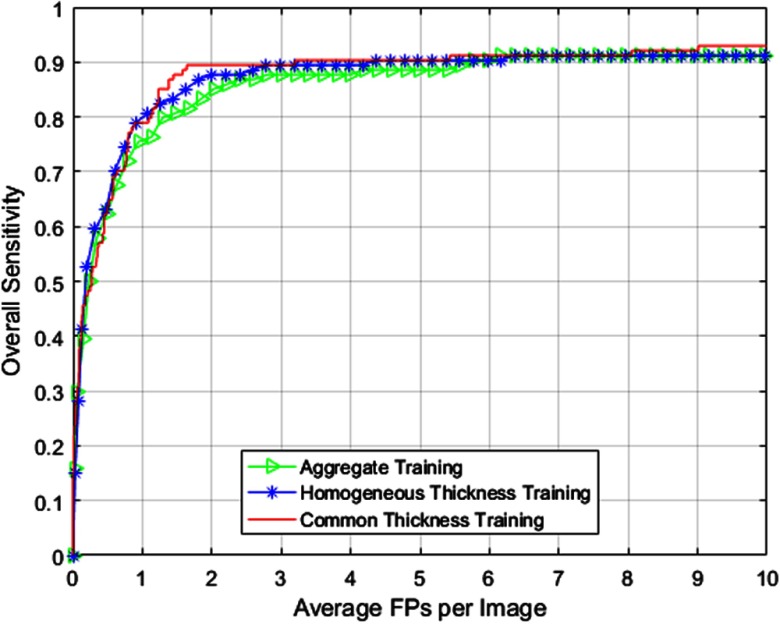
FROC curves comparing overall CAD performance using different training methods for 2.5-mm testing dataset utilizing the composition provided in Table 5.

Figure 8 and Table 6 present the overall CAD performance for the three different training methods with 100 cases acquired at 2.5 mm being utilized for testing. Note that 2.5-mm testing cases are not resampled for any classification method.

**Table 6:** Overall CAD performance comparison using different training methods for 2.5-mm testing dataset.

Training method	Candidate detector sensitivity (before classification)	Number of features selected for classification	Overall CAD performance AUC (0 to 10 FPs)	95% Confidence AUC (0 to 10 FPs) interval	ANODE score
Aggregate	96.49	13	8.57	8.45±0.50	0.705
Homogeneous thickness	96.49	12	8.68	8.61±0.56	0.718
Common thickness	**96.49**	**12**	**8.74**	8.66±0.47	**0.723**

Note: Bold values represent the best performance among the compared methods.

### 5.4 Experiment Based on the Entire LUNA16 Dataset

**Fig. 9:**
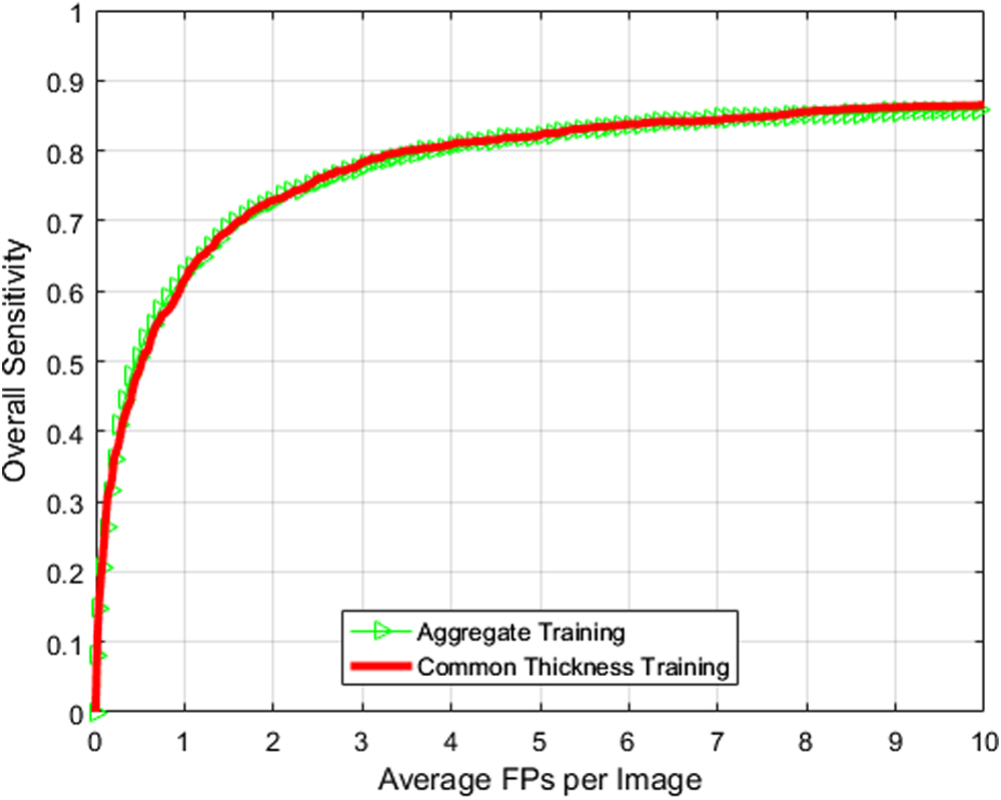
FROC curves comparing aggregate and common thickness training methods for the entire LUNA16 dataset.

In this section, we present results comparing the aggregate and common thickness methods of training for the entire suite of 888 cases from the LUNA16 grand challenge.^39^ The homogeneous thickness training method is not performed in this experiment due to insufficient training data at each thickness value. Overall CAD performance is analyzed based on 10-fold validation. Note that we perform SFS based on each combination of training folds, i.e., we perform 10 different SFS processes for 10 different training sets. Cases chosen for each fold are the same as provided in the LUNA16 grand challenge.^38^^,^^39^ Figure 9 shows the overall FROC curve obtained using the aggregate and common thickness methods of training. Overall AUC values along with their confidence intervals are provided in Table 7. Results clearly indicate that performance of the common thickness training method is comparable with the aggregate method of training. However, performance can be achieved in significantly less time using the common thickness method of training.

**Table 7:** Overall CAD performance comparison using different training methods for the entire LUNA16 dataset.

Training method	Candidate detector sensitivity (before classification)	Overall CAD performance AUC (0 to 10 FPs)	95% Confidence AUC (0 to 10 FPs) interval	ANODE score
Aggregate	90.36	7.75	7.77±0.19	0.596
Common thickness	**92.38**	**7.75**	7.78±0.08	**0.597**

Note: Bold values represent the best performance among the compared methods.

## 6 Discussion

Several papers have addressed the study of CAD systems as a function of slice thickness, but no research work that we are aware of has been implemented on the newly discovered LUNA16 dataset. We studied the performance using many cases for a wide range of thickness utilizing the same set of nodule cue points marked by the radiologists at native thickness. Utilizing cases with slice thickness equal to slice spacing for the study helped us achieve simulated higher thickness by a simple downsampling process. Performance was studied at 1.25, 2.5, 5, and 10 mm. This study helped us in determining the thickness for optimal CAD performance in terms of accuracy, memory consumption, and computational speed.

We also addressed the issue of managing nonhomogeneous training data in terms of slice thickness. We analyzed the performance of three different training methodologies to obtain the best CAD performance with the available training data. We studied these methods under different testing conditions, i.e., CT scans natively acquired at 1.25 and 2.5 mm, respectively. Later, we also studied the performance for the entire suite of 888 cases in the LUNA16 dataset. Analyzing the performance of training methods for a diverse set of testing data helped us identify the best method depending on the test set in question. Studying the confidence intervals helped us analyze the statistical significance and variance for each training method.

Our CAD system adopted in this research^8^ produced state-of-the-art performance in ANODE 2009.^42^ Most of the CAD systems presented in the literature adopt a similar approach that includes lung segmentation, candidate detection, feature computation, and classification for lung nodule detection. We believe our findings are relevant to this broad class of CAD systems. We acknowledge the fact that our results and analysis are based on the performance of a specific CAD system and may vary using other CAD systems.

## 7 Conclusions

In this paper, we have presented two thickness-based studies for CAD of lung nodules in CT scans. First, the study presented the performance of the CAD system at various thickness levels. FROC results presented in Fig. 5 and Table 2 indicate that the CAD system provides comparable performance at native thickness and simulated thickness of 2.5 mm. In fact, the CAD system achieves good performance at a much faster rate (2×) with reduced memory consumption when downsampled to a simulated thickness of 2.5 mm. However, classification performance deteriorates considerably when downsampled further than 2.5 mm. Our experimental results suggest that, with the same amount of data across various thickness values (1.25, 2.5, 5, and 10 mm), 2.5 mm is the most effective in terms of accuracy, dosage level, computation, and memory consumption.

Second, we presented results comparing CAD performance using three training methods for nonhomogeneous data. Figures 7–9 indicate that the common thickness method of training (at 2.5 mm) provides the best results for all sets of testing data studied in this paper. Tables 4, 6, and 7 indicate that AUC and ANODE score follow the same trend in terms of performance. Confidence intervals presented in Tables 4, 6, and 7 indicate that the common thickness method is more consistent in terms of performance when compared with other methods. The common thickness method helps in maintaining the homogeneity among the cases (training and testing) and in utilizing all the cases available for training. This performance is closely followed by the aggregate method of training, albeit using increased memory and more computation time. The homogeneous thickness method of training could be utilized when there are sufficient training cases that match with the thickness of the testing cases.
